# Apollo Lunar Astronauts Show Higher Cardiovascular Disease Mortality: Possible Deep Space Radiation Effects on the Vascular Endothelium

**DOI:** 10.1038/srep29901

**Published:** 2016-07-28

**Authors:** Michael D. Delp, Jacqueline M. Charvat, Charles L. Limoli, Ruth K. Globus, Payal Ghosh

**Affiliations:** 1 Department of Nutrition, Food and Exercise Sciences, Florida State University, Tallahassee, FL 32306, USA; 2 Wyle Science, Technology and Engineering Group, Johnson Space Center, Houston TX 77058, USA; 3 Department of Radiation Oncology, University of California Irvine, Irvine, CA 92697, USA; 4 Space Biosciences Division, NASA Ames Research Center, Moffett Field, CA 94035, USA.

## Abstract

As multiple spacefaring nations contemplate extended manned missions to Mars and the Moon, health risks could be elevated as travel goes beyond the Earth’s protective magnetosphere into the more intense deep space radiation environment. The primary purpose of this study was to determine whether mortality rates due to cardiovascular disease (CVD), cancer, accidents and all other causes of death differ in (1) astronauts who never flew orbital missions in space, (2) astronauts who flew only in low Earth orbit (LEO), and (3) Apollo lunar astronauts, the only humans to have traveled beyond Earth’s magnetosphere. Results show there were no differences in CVD mortality rate between non-flight (9%) and LEO (11%) astronauts. However, the CVD mortality rate among Apollo lunar astronauts (43%) was 4–5 times higher than in non-flight and LEO astronauts. To test a possible mechanistic basis for these findings, a secondary purpose was to determine the long-term effects of simulated weightlessness and space-relevant total-body irradiation on vascular responsiveness in mice. The results demonstrate that space-relevant irradiation induces a sustained vascular endothelial cell dysfunction. Such impairment is known to lead to occlusive artery disease, and may be an important risk factor for CVD among astronauts exposed to deep space radiation.

The human experience with spaceflight has shown that space exploration comes with various health risks^1^,^2^,^3^. As humans contemplate manned missions to Mars or prolonged habitation on the Moon, these health risks will rise as the duration of spaceflight increases and as travel goes beyond the Earth’s protective magnetosphere^4^,^5^. During such interplanetary travel, astronauts will be exposed to multiple sources of ionizing radiation, including galactic cosmic rays, solar particle events, and trapped radiation in the Van Allen belts. Protons are the most abundant type of radiation in space, while heavier high atomic number and energy (HZE) ions such as ^56^Fe produce complex tissue damage to molecules such as DNA that challenges cellular repair and recovery^6^,^7^,^8^,^9^.

Recent epidemiological studies have demonstrated increasing risk for cardiovascular disease resulting from ionizing radiation exposure^10^,^11^,^12^. However, these estimates are largely derived from low linear energy transfer (LET) radiation exposures such as X-rays or gamma rays, which have fundamentally different properties from charged HZE particles comprising the galactic cosmic rays. HZE ions, for example, produce greater adverse effects on cellular physiology through increased genetic alterations and perturbations to redox metabolism, leading to persistent elevations in oxidative stress^13^,^14^,^15^.

Despite the potential to be more biologically damaging, the long-term effect of space radiation on cardiovascular health has received little attention^16^,^17^,^18^. Therefore, the primary purpose of the present study was to determine whether mortality rates due to cardiovascular disease (CVD) and other causes of death differ in (1) non-flight astronauts who never flew orbital missions in space, (2) astronauts who flew only in low Earth orbit (LEO), and (3) Apollo lunar astronauts, the only humans to have traveled outside of the Earth’s geomagnetic field and into deep space.

A heightened risk for CVD among flight astronauts could be due to the direct effects of space radiation and weightlessness on the arterial vasculature. Using ^56^Fe ion irradiation and hindlimb unloading (HU), a terrestrial rodent model that simulates the irradiation and musculoskeletal unloading that occurs during deep space travel^19^,^20^, the secondary purpose of this study was to determine whether simulated space radiation and weightlessness independently and jointly produce long-lasting vascular dysfunction that could lead to the development of atherosclerotic cardiovascular disease.

## Results

### Group Characteristics and Proportional Mortality Rates

The group of all flight astronauts was comprised of 5 females and 37 males, of which the LEO astronaut subgroup contained 5 females and 30 males and the Apollo lunar astronaut subgroup was comprised of 7 males. The non-flight astronauts consisted of 3 females and 32 males. Differences in the mean age at the time of selection and the time of death between all flight and non-flight astronauts or among LEO, lunar and non-flight astronauts were not significant (Table 1). Although the duration of Apollo missions ranged from 6.0–12.6 days, several of the lunar astronauts also logged time on Mercury, Gemini and Skylab missions, resulting in a total time in space of ~15 days. The total time in space and the number of missions flown did not differ between LEO and Apollo lunar astronauts (Table 1).

**Figure 1:**
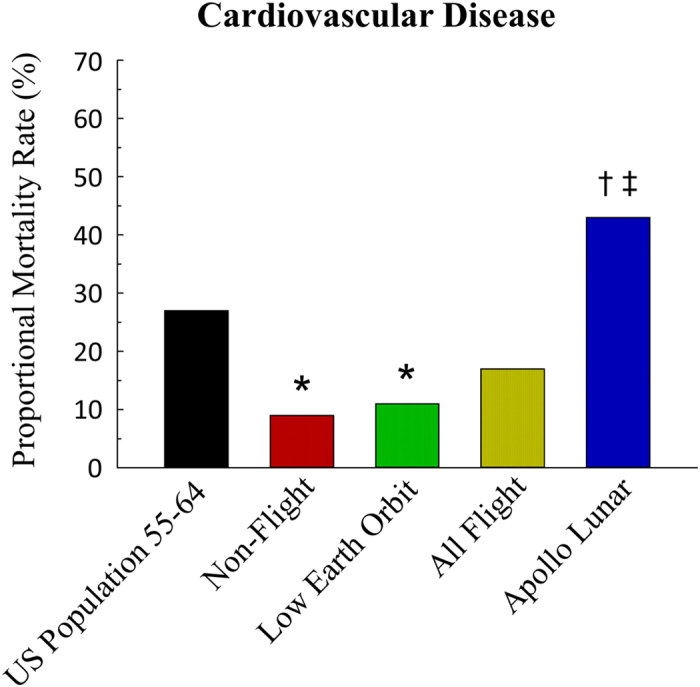
The proportional mortality rate due to cardiovascular disease in the United States among individuals age 55–64 years, non-flight astronauts, astronauts that flew only low Earth orbit missions, all flight astronauts, and Apollo astronauts that flew missions to the Moon. *Significantly different from the US population 55–64 years of age at the time of death, P ≤ 0.05. ^**†**^Significantly different from the non-flight astronaut group, P ≤ 0.05. ^**‡**^Significantly different from the low Earth orbit astronaut group, P ≤ 0.1.

The proportional mortality rate due to CVD for all flight astronauts was not different from that in the non-flight astronauts (Fig. 1). However, when looking at the subgroup analysis the number of deaths due to CVD in Apollo lunar astronauts was almost five times greater than that in the non-flight astronauts (Fig. 1) and four times higher than in LEO astronauts. There were no differences between LEO and non-flight astronauts. All flight and non-flight astronauts that died from cardiovascular-related causes were male.

There were no differences in the proportional mortality rates due to cancer between flight (both LEO and lunar) and non-flight astronauts (Table 2). Deaths due to accidents were not different between flight or LEO and non-flight astronauts, but the proportional mortality rates of lunar astronauts due to accidents was lower than that of non-flight astronauts (Table 2). Proportional mortality rates for other causes of death were not different between flight, LEO or lunar and non-flight astronauts (Table 2).

In 2013, the latest year for cause-specific mortality data in the US, there were 338,127 deaths that occurred in the US among individuals in the 55–64 age range^21^. Relative to this 55–64 year old US population, the proportional mortality rate due to CVD was lower in the non-flight astronauts and LEO astronauts (Fig. 1). Cancer proportional mortality rate was not different between the US population and both flight and non-flight astronauts (Table 2). Proportional mortality due to accidents was higher in both flight and non-flight astronauts compared with the US population (Table 2). Finally, for all other causes of death there was a lower proportional mortality for flight, LEO and non-flight astronauts compared with the US reference population, but no differences between lunar astronauts and the 55–64 year old population (Table 2).

### Animal and Vessel Characteristics

Following a 6–7 month recovery period from exposure to simulated weightlessness and space radiation, total body mass, muscle masses and gastrocnemius muscle feed artery characteristics were not different among groups (Table 3).

### Vasodilator Responses

**Figure 2:**
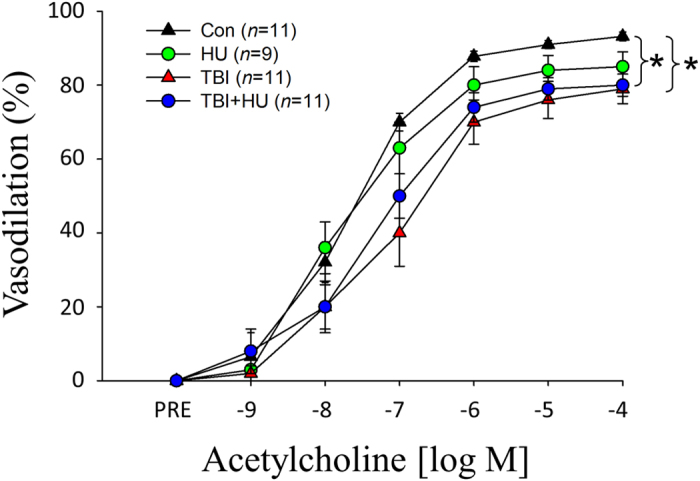
Effects of hindlimb unloading (HU) and total body irradiated (TBI), individually and combined (TBI+HU) on ACh-mediated vasodilator responses in gastrocnemius muscle feed arteries. Values are mean ± SE. *n* = the number of animals studied. *Denotes significant dose by group interaction between groups, P ≤ 0.05; TBI and TBI+HU group responses are different from that of control (Con).

**Figure 3:**
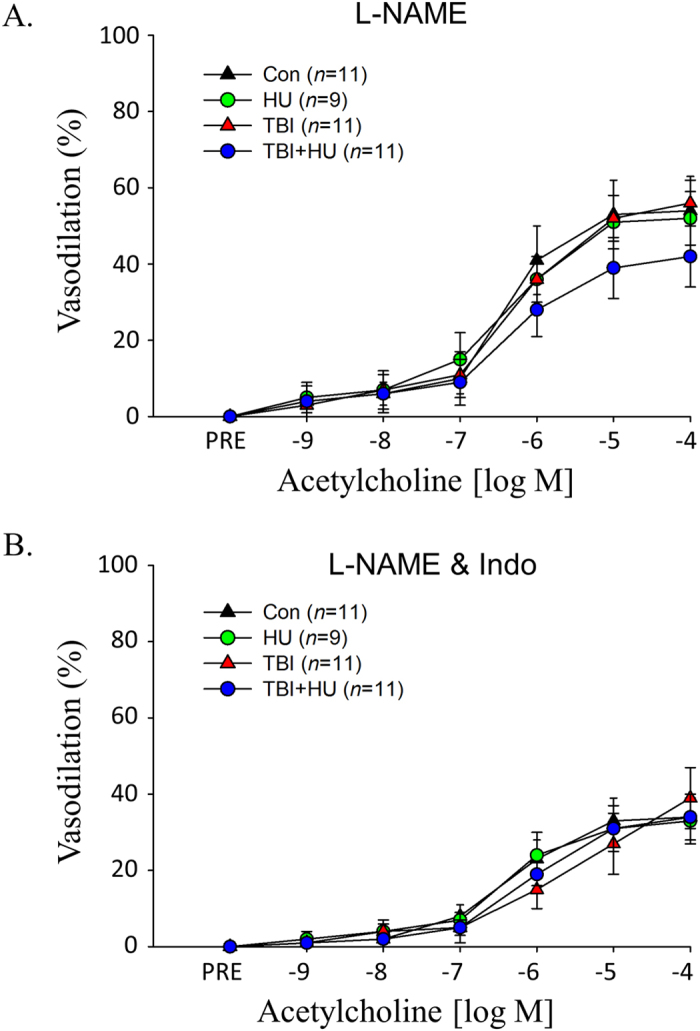
Effects of HU, TBI, and TBI+HU on ACh-mediated vasodilator responses in the presence of (**A**) the NOS inhibitor L-NAME, and (**B**) L-NAME and the COX inhibitor indomethacin (Indo), in gastrocnemius muscle feed arteries. Values are mean ± SE. *n* = the number of animals studied. ACh-mediated vasodilator responses are not different among groups.

**Figure 4:**
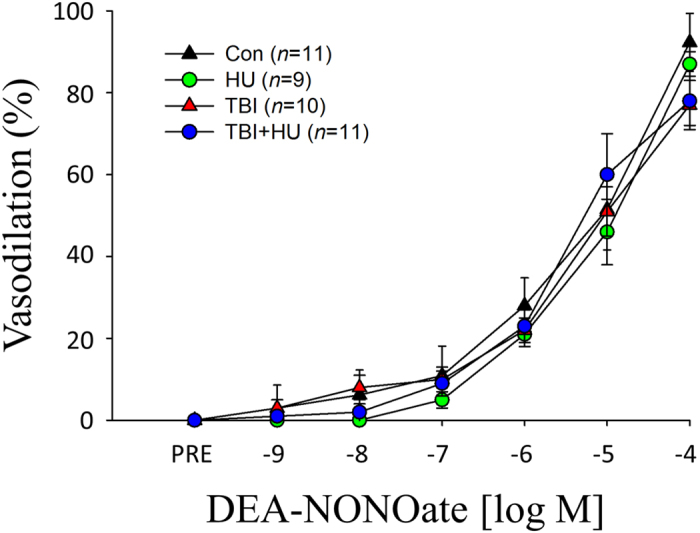
Effects of HU, TBI, and TBI+HU on Dea-NONOate-induced vasodilator responses in gastrocnemius muscle feed arteries. Values are mean ± SE. *n* = the number of animals studied. Vasodilator responses are not different among groups.

There was a significant acetylcholine (ACh) dose by group interaction between the control (Con) and total body irradiated (TBI) groups and the Con and total body irradiated plus hindlimb unloaded (TBI+HU) groups (Fig. 2). The nitric oxide synthase (NOS) inhibitor N^G^-nitro-l-arginine methyl ester (L-NAME) reduced ACh-mediated vasodilation in all groups and abolished treatment-associated differences among groups (Fig. 3A). The combined NOS and cyclooxygenase (COX) inhibition further decreased vasodilator responses with no differences among groups (Fig. 3B). There was no significant dose by group interaction for endothelium-independent vasodilation with Dea-NONOate among groups (Fig. 4).

### Vasoconstrictor Responses

There were no differences in vasoconstriction mediated by KCl (Supplemental Fig. 1A) or phenylephrine (PE) (Supplemental Fig. 1B) among groups. Both active myogenic vasoconstriction (Supplemental Fig. 2A) and passive pressure-diameter responses (Supplemental Fig. 2B) were also not different among groups.

### Protein Expression

**Figure 5:**
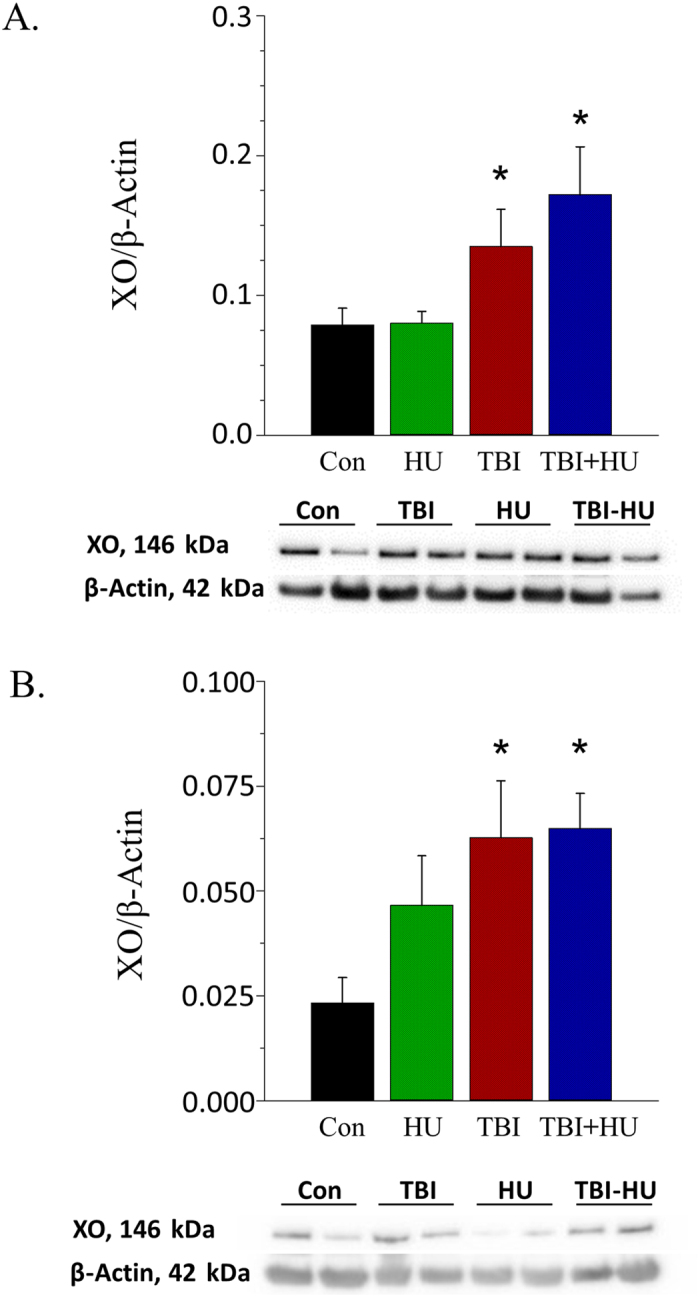
Effects of HU, TBI and TBI+HU on xanthine oxidase (XO) protein levels in (**A**) gastrocnemius muscle feed arteries and (**B**) coronary arteries. Values are mean ± SE. *Denotes significant difference from Con group.

Levels of the pro-oxidant protein xanthine oxidase (XO) were greater in gastrocnemius feed arteries (Fig. 5A) and coronary arteries (Fig. 5B) from TBI and TBI+HU mice, while endothelial NOS (eNOS), superoxide dismutase-1 (SOD-1) and NADPH oxidase-2 (NOX-2) protein content were not different among groups in gastrocnemius feed arteries (Supplemental Fig. 3) and coronary arteries (Supplemental Fig. 4).

## Discussion

Life on Earth is insulated from the harmful effects of galactic cosmic rays and solar particle events through particle deflection by the Earth’s magnetosphere and shielding by Earth’s atmosphere. With the exception of the Apollo lunar missions, manned spaceflight has taken place exclusively in LEO where atmospheric protection from space radiation is essentially absent, but protection by the Earth’s geomagnetic field is present. Under these conditions, it has been broadly assumed that missions in LEO or short excursions to the Moon would not increase the long-term risk for CVD among astronauts^16^,^18^. Using non-flight astronauts as a comparison group, the data show that this group had a mortality rate of approximately 9% due to CVD (Fig. 1). The mortality rate for CVD among all US flight astronauts, including both LEO and Apollo lunar astronauts, was not different than that in the non-flight astronauts. However, when considered as a separate group, the Apollo lunar astronauts, the only group of humans to have traveled outside of the Earth’s protective magnetosphere, demonstrate a higher mortality rate due to CVD compared to both the cohort of astronauts that did not travel into space, as well as astronauts who remained in LEO (Fig. 1). These data suggest that human travel into deep space may be more hazardous to cardiovascular health than previously estimated.

Astronaut mortality has been reported in four previous studies. In the first by Peterson *et al*.^22^, it was reported that of the 20 deceased US astronauts from 1959–1991 the causes of death were due to circulatory disease (10%), cancer (5%), accidents (80%) and other causes (5%). Using the US population as the reference group, it was found that mortality due to CVD was significantly lower in astronauts and accidental deaths were significantly higher^22^. A subsequent study by Hamm *et al*.^23^ focused solely on cancer-specific mortality among astronauts through 1995, and included an additional reference group of Johnson Space Center employees. This comparison group was part of a Longitudinal Study of Astronaut Health initiative by the National Aeronautics and Space Administration (NASA) to better assess the occupational health risks of astronauts, since the health characteristics of astronauts and the overall environment in which they train at Johnson Space Center were deemed to be different from that of the general US population^24^. Results from this study indicated that there was not a significant difference in cancer mortality between astronauts and Johnson Space Center employees or Texas residents living in the area surrounding Johnson Space Center^23^. In a follow-up study by Hamm *et al*.^25^, cause-specific mortality rates beyond just cancer were examined. The only significant difference found was a higher number of accidental deaths among astronauts (69%) relative to Johnson Space Center employees (14%). Finally, in the most recent study published by Reynolds and Day^26^, the cause-specific mortality rates from 1980–2009 were reported in astronauts and residents of Harris County, Texas, where Johnson Space Center is located. The results indicated a lower risk of death due to CVD and cancer in astronauts versus Harris County residents, and a higher risk of death due to accidents^26^. Collectively, these studies indicate that the risk of death due to chronic diseases appears to be lower in astronauts, particularly those involving CVD^22^,^26^.

The current analysis of mortality rates among astronauts differs from these previous studies in several important ways. The first is the reference population used to gauge the significance of cause-specific mortality rates among astronauts. The biomedical characteristics of astronauts are very different from individuals in the general population. According to the Review of NASA’s Longitudinal Study of Astronaut Health by the Institute of Medicine^24^, astronauts have substantially higher incomes, levels of education, general fitness, and lifelong access to medical care, all of which are factors known to contribute to high levels of health and well-being. With such large baseline differences between astronauts and comparison groups, it is difficult to ascertain what specific impact spaceflight might have on astronaut health. For this reason, the Institute of Medicine recommended in 2004 that astronauts who have never flown in space be used as a reference population^24^; the present study is the first to incorporate non-flight astronauts as a comparison group. Consequently, current results demonstrate that previous conclusions suggesting the risk of death due to CVD is lower among flight astronauts^22^,^26^ are no longer tenable.

The second unique feature of the present study is that it is the first to examine the long-term mortality risks of spaceflight in LEO and deep space. Exposure to charged particles comprising the galactic cosmic rays in deep space has the potential to elicit a number of complications in biological tissue. Recent work in rodents and cell culture has highlighted the potentially harmful effects of such exposures on the cardiovascular system^20^,^27^,^28^,^29^,^30^, which may translate to astronauts engaged in deep space expeditions.

While traveling from LEO to the Moon and back, the Apollo lunar astronauts traversed regions of geomagnetically trapped electrons and protons known as the Van Allen belts and, depending on the duration of their mission and the specific activities in which they were engaged (e.g., lunar surface and intravehicular Command and Lunar Module activities), were continuously subjected to varying levels of high-energy cosmic rays^31^. Fortunately, there were no major solar particle events during any of the Apollo missions. The lunar astronauts also experienced a visual phenomenon of light flashes (~17/hour) when the spacecraft was dark, and tests indicated that the flashes were the result of HZE cosmic rays traversing the retina^31^,^32^.

Interactions of the galactic cosmic rays with the spacecraft hull will have a large impact on the radiation exposure of astronauts. Charged particles traversing the hull or “shielding” of the ship will incur nuclear interactions that depend on the composition and thickness of the hull material. These interactions will result in fragmentation products and particles of reduced energy but higher LET that contribute to the radiation dose within the spacecraft. The average radiation dose for the seven deceased Apollo crew was 0.59 ± 0.15 cGy (range 0.18–1.14 cGy)^31^. If we assume representative shielding scenarios (10 g/cm^2^) for the Apollo Command Module and radiation quality factors drawn from the most recent International Commission on Radiological Protection^33^, then the average radiation dose from the galactic cosmic rays to the Apollo astronauts would be approximately 0.295 cGy, or roughly half the total dose during their lunar excursions^9^. Using similar assumptions, astronauts in LEO would receive 50–100 mSv over a 6–12 month stay, of which the galactic cosmic rays would account for approximately two-thirds of this total dose^9^. Thus, given their mean mission duration of 15.6 days, the deceased LEO astronauts would receive approximately 0.29 cGy, a galactic cosmic ray dose very similar to the Apollo lunar astronauts.

Despite virtually identical estimates for galactic cosmic ray exposure, the mortality rate of LEO astronauts for CVD is significantly lower than in Apollo lunar astronauts. Furthermore, LEO astronauts do not exhibit significant differences in mortality compared to non-flight astronauts. Several factors may account for this apparent paradox. First, lunar and LEO galactic cosmic ray dose estimates were made using certain assumptions that are constantly being revised. For instance, if actual shielding levels for the Apollo missions were less than 10 g/cm^2^, calculated lunar galactic cosmic ray doses may be underestimates. Second, activities on the lunar surface and inside the lightly shielded Lunar Module may also include dose contributions from scattered albedo neutrons, which are relatively insignificant inside a spacecraft. And finally, lunar and deep space exposures will include dose contributions from less energetic and lighter particles. For astronauts in LEO, the relative contribution of particles with energies below the geomagnetic cutoff is lower since they will be deflected by the Earth’s magnetosphere. While it remains uncertain whether differences in absorbed dose profiles can account for the elevated lunar CVD mortality rates reported here, it is equally difficult to disregard this possibility. As a result, these findings highlight the potential adverse impact of charged particles and their unique microdosimetric properties on post-mitotic cellular structures responsible for maintaining longer-term cardiovascular health.

The possibility of long-term degenerative effects of deep space travel on cardiovascular function has not been well described or substantiated. Only in the last decade, when multiple spacefaring nations and corporate entities have announced plans to embark on manned exploratory missions to Mars and prolonged habitation on the Moon, has biomedical research been directed towards identifying possible CVD risks associated with the deep space radiation environment. Consequently, there is limited information available on the effects of charged particle HZE radiation on the cardiovascular system.

Results from the present study address the question of possible long-lasting interactive effects of simulated weightlessness and space radiation on vascular function. To experimentally address this question, vascular responses of resistance arteries were determined 6–7 months after the cessation of a 14-day hindlimb unloading treatment, a 1 Gy ^56^Fe irradiation treatment, or a treatment consisting of a combination of the two. Given that the average life-span of male C57BL/6 mice is 878 ± 10 days^34^, the 6–7 month period represents approximately 23% of animals’ life or roughly the equivalent of 18–20 years in humans. The data show that hindlimb unloading alone had no persistent effect to significantly diminish endothelium-dependent vasodilation, while HZE irradiation alone and in combination with unloading impaired endothelium-dependent vasodilation (Fig. 2) through the NO signaling mechanism (Fig. 3). This decrement in NO signaling appears to be mediated primarily through greater NO scavenging by reactive oxygen species, as evidenced by higher vascular protein content (Fig. 5A,B)^20^,^27^ and activity^27^ of XO in peripheral and coronary arteries. These data indicate that the long-lasting effects of simulated weightlessness and space radiation on vascular endothelial function are the result of the radiation exposure and are not due to an interaction with weightlessness. As dysfunction of the vascular endothelium is central to the pathogenesis of vascular disease^35^,^36^, such adverse arterial effects could lead to the development of occlusive arterial diseases, including myocardial infarction and stroke.

Although results from the present study provide new evidence that even short-term spaceflight beyond the Earth’s protective magnetosphere may have adverse effects on CVD mortality, there are limitations to consider. First, the sample size for cause-specific deaths among lunar astronauts is small. Therefore, caution must be used in drawing definitive conclusions regarding specific health risks. Second, although deep space radiation seems a likely cause underlying the higher proportional mortality rate due to CVD in Apollo astronauts, it remains unknown what specific factor(s) in the space environment is responsible. And third, although the HZE irradiation used in the animal studies was selected to mimic that which deep space travelers might encounter, the absorbed dose (1 Gy) and the dose rate (single exposure, 10cGy/min) would be higher and faster than that experienced by the Apollo lunar astronauts^31^. It would be more representative of the space environment to have the radiation exposure occur at a lower dose rate and over an extended time period. Unfortunately, that type of exposure paradigm using ^56^Fe ions is not currently feasible at Brookhaven National Laboratory. Despite these limitations, results from these animal studies demonstrate that space relevant irradiation induces long-lasting vascular dysfunction of the type known to presage the development of atherosclerotic cardiovascular disease^35^,^36^.

In summary, results from the present study reveal that Apollo lunar astronauts have a significantly higher mortality rate due to CVD than either the cohort of astronauts who never flew an orbital space mission or astronauts who never flew beyond LEO (Fig. 1). Moreover, the CVD mortality for lunar flight astronauts was higher than that in the age-matched US population, although this difference was not statistically significant. These findings suggest that in spite of the “healthy worker” effect, short-duration deep space travel by this highly educated, trained and physically fit group results in a significantly elevated risk of death from CVD. The major environmental factor that would appear to underlie this phenomenon is deep space radiation. Estimates indicate that the dose of galactic cosmic ray irradiation to which LEO and lunar astronauts were exposed were not greatly different. However, qualitative differences in the absorbed dose profiles resulting from the effect of the Earth’s magnetosphere to deflect less energetic and lighter galactic cosmic ray particles away from Earth may account for the lower CVD mortality rate among LEO astronauts. Animal studies also indicate that while simulated weightlessness and space-relevant irradiation interact to induce early impairment of endothelium-dependent vasodilation^20^, the only sustained vascular endothelial cell dysfunction is that mediated by exposure to HZE particles and not by simulated weightlessness (Fig. 2). If such results translate to the human condition, then long-term dysfunction of the vascular endothelium induced by charged HZE particles could be a major contributor to the development of atherosclerotic cardiovascular disease in astronauts.

## Methods

### US Astronaut Mortality Rate Study

This study consisted of two distinct populations, the deceased astronaut population and the deceased US National Population ages 55–64. A record of deceased US NASA astronauts through 2015 was obtained from NASA Johnson Space Center Lifetime Surveillance of Astronaut Health and NASA website (http://www.jsc.nasa.gov/Bios/astrobio_former.html). Records of other NASA-US Air Force astronauts in the X-15, X-20 and Manned Orbital Laboratory programs were obtained from program summaries^37^. Deceased US astronauts were further grouped into flight (including LEO and Apollo lunar subgroups) or non-flight astronaut samples based on their flight activities; this allowed cause of death comparisons between the flight and non-flight groups.

### US Astronaut Flight Sample

Deceased flight astronauts (*n* = 43) flew on orbital missions as part of the Mercury, Gemini, Apollo, Apollo-Soyuz, Skylab and Space Shuttle programs and included pilots, mission commanders, mission specialists and payload specialists. Deceased flight astronauts were further subdivided into two groups, those having flown missions in low Earth orbit (LEO, *n* = 36) and those having flown missions beyond LEO as part of the Apollo lunar missions (*n* = 7). Astronauts that perished in the Apollo 1 and STS-51L Challenger accidents but had flown on previous missions (*n* = 6) were classified as flight astronauts. Those US astronauts that perished in the STS-107 Columbia accident were classified as flight astronauts since all achieved orbital flight.

### US Astronaut Non-Flight Comparison Sample

The first comparison group consisted of deceased astronauts who never flew in space or flew only suborbital flights (*n* = 41). These individuals included astronauts that completed astronaut training but were never assigned to a specific mission, were assigned to a subsequently cancelled missions, died prior to flying their assigned mission, flew the NASA-US Air Force’s X-15 rocket-powered aircraft, or were designated to fly on the US Air Force’s X-20 Dyna-Soar space plane or Manned Orbiting Laboratory (MOL). Astronauts that flew the X-15 aircraft never attained orbital spaceflight, and individuals assigned to the X-20 Dyna-Soar and MOL never flew in space as part of these programs due to their cancelation. Of the thirty-one X-15, X-20 and MOL astronauts, nine subsequently flew orbital missions as part of the Apollo and Space Shuttle programs. Four of the nine are now deceased and included in the flight group; three are in the LEO group and one is in the Apollo lunar group. Astronauts who perished in the Apollo 1 and STS-51L Challenger accidents who were first-time flyers (*n* = 4) were categorized as non-flight astronauts for the purpose of this analysis due to not having achieved orbital flight.

### US National Population Comparison Sample

A second comparison population consisted of the general US population. The total number of deaths in 2013 of those dying between 55–64 years of age and the number of deaths from this population subgroup due to CVD, cancer, accidents, and all other causes were used for comparison with that of the flight (including LEO and Apollo lunar subgroups) and non-flight astronaut groups. This population subgroup from the general population was used because the 55–64 year age group most closely approximates the ages of the flight and non-flight astronauts at their time of death (Table 1).

### Vital Statistics

Cause of death was determined from death certificates in all Apollo lunar astronauts, 97% of LEO astronauts, and 49% of non-flight astronauts. In cases where death certificates were not available for review, cause of death was obtained from official NASA biographical sketches (http://www.jsc.nasa.gov/Bios/), NASA’s Astronaut Fact Book, obituaries, and other biographical records. The specific cause of death for one LEO astronaut and 6 non-flight astronauts could not be determined from any of the above sources; these astronauts were not included in the analysis for a final sample of 42 flight astronauts and 35 non-flight astronauts.

Cause of death was categorized into four main categories: Cardiovascular Disease, Cancer, Accident, and All Other Causes. Deaths due to heart failure, myocardial infarction, stroke, brain aneurysm, or blood clots were classified as CVD. Deaths attributed to any form of malignant neoplasms were categorized as cancer. Deaths due to unintentional injuries, including orbiter destruction and plane, automobile, motorcycle, boat and bicycle accidents, were categorized as accidents. Any other causes of death were designated in the All Other Causes category and included illnesses such as pneumonia and pancreatitis. All-cause and cause-specific mortality rates for the US general population 55–64 years old in 2013 were obtained from the Centers for Disease Control and Prevention’s National Center for Health Statistics^21^.

### Animal Studies

All experimental procedures were approved by the Institutional Animal Care and Use Committees at Florida State University, NASA and Brookhaven National Laboratory, conforming to the U.S. National Institutes of Health (NIH) *Guide for the Care and Use of Laboratory Animals* (Eighth edition, 2011).

Forty-four male C57BL/6 mice (Jackson Laboratory, Bar Harbor, ME), 16 weeks of age, were individually housed at the Brookhaven National Laboratory animal facility at Long Island, New York. Animals were maintained in a controlled environment (12:12 hour light-dark cycle, 24 ± 2 °C) and provided food and water *ad libitum*. Mice were randomized by body mass to one of four groups: control (Con, *n* = 11), hindlimb unloaded (HU, *n* = 11), total body irradiated (TBI, *n* = 11), and the combined TBI and HU (TBI+HU, *n* = 11). One week after the conclusion of the unloading treatment for the HU and TBI+HU groups, the mice in all four groups were shipped to Florida State University, individually housed in the animal vivarium under controlled environmental conditions (12:12 hour light-dark cycle, 24 ± 2 °C) and provided food and water *ad libitum*.

### Hindlimb Unloading

Mice were hindlimb unloaded via tail traction for 14 days according to the methods of Morey-Holton *et al*. as previously described^19^,^20^,^38^. After the 14-day unloading treatment, animals were released from the unloading apparatus to move freely in standard cages for 6–7 months until the time to conduct the vascular experiments. Control mice were individually housed in their normal cage environment.

### Whole-Body Irradiation

Mice were exposed to a single dose of radiation consisting of 1 Gy of ^56^Fe ions (600 MeV/nucleon, LET 150 keV/μm in water) at a dose rate of 10cGy/min at the NASA Space Radiation Laboratory beamline at Brookhaven National Laboratory. Irradiation of TBI+HU mice took place 3 days after the initiation of hindlimb unloading while the animals remained unloaded. Sham-irradiated mice were handled in an identical fashion but were not irradiated (0 Gy).

### Isolated Microvessel Studies

Experiments were conducted on 2 mice per day over a 5-wk period. These studies commenced after at least 6 months from the time the HU mice were released from the unloading treatment. Animals from each of the four groups were randomly selected for experimentation over the 5-wk experimental period, and one mouse from each group was studied every other experimental day. Mice were anesthetized with isoflurane (5%/O_2_) and euthanized by excision of the heart. Gastrocnemius muscle feed arteries running along the superficial white portion of the gastrocnemius muscles from the left and right hindlimbs were isolated and either cannulated and prepared for *in vitro* experimentation or frozen in liquid N_2_ and stored at −80 °C for determination of protein content as previously described^20^,^38^. Additionally, the left anterior descending artery and branches off this artery (~90–220 μm inner diameter) were dissected free of the surrounding myocardium, snap frozen in liquid N_2_ and stored at −80 °C for determination of protein content.

### Evaluation of Vasomotor Properties

In one set of studies, vasodilator responses of feed arteries to the endothelium-dependent vasodilator acetylcholine (ACh, 10^−9^–10^−4^ M) and endothelium-independent vasodilator Dea-NONOate (10^−9^–10^−4^ M) were assessed as previously described^20^,^38^. ACh-induced vasodilation was also evaluated following incubation with nitric oxide synthase (NOS) inhibitor N^G^-nitro-l-arginine methyl ester (L-NAME, 10^−5^ M) and following incubation with the combination of L-NAME with COX inhibitor indomethacin (10^−5^ M) as previously reported^20^,^38^. Maximal diameter and medial wall thickness were determined after a 1-hr incubation period in calcium-free physiological saline buffer solution (PSS) with 10^−4^ M sodium nitroprusside (SNP) to allow complete smooth muscle cell relaxation.

In a second set of vessels, vasoconstrictor responses were assessed through non-receptor (KCl, 10–100 mM), myogenic (increasing intraluminal pressure from 0 cm H_2_O up to 140 cm H_2_O, in 20 cm H_2_O increments), and receptor [α_1_-adrenoreceptor agonist phenylephrine (PE, 10^−9^–10^−4^ M)] mechanisms as previously described^20^,^39^. Passive pressure-diameter responses were recorded in a similar fashion to that of the active myogenic response after vessels were incubated in calcium-free PSS with 10^−4^ M SNP for 1 hr.

### Immunoblot Analysis

eNOS, SOD-1, XO and NOX-2 protein content in gastrocnemius feed arteries and coronary arteries were assessed via immunoblot analysis. Arteries were isolated, snap frozen and processed as previously described^20^,^40^. Primary antibody dilutions were as follows: eNOS (1:150, BD Transduction #610296), Cu/Zn SOD-1 (1:8000, Enzo Life Sciences ADI-SOD-100-F), XO (1:1000, Abcam #109235), NOX-2 (1:1000, Abcam #129068) and β-actin (1:2000, Cell Signaling #4967). Differences in loading were normalized by expressing all data as relative densitometry units of the protein of interest versus β-actin.

### Statistical Analysis

Proportional mortality rates were calculated as the number of deaths from a particular cause divided by the total number of deaths for that particular group or sub-group. The significance of differences in cause-specific deaths between groups was assessed with Fisher’s exact probability test. Due to this test being considered extremely conservative^41^, a value of P ≤ 0.10 was considered statistically significant^42^,^43^. Differences in age at the time of death and other astronaut characteristics were assessed with a one-way ANOVA and Fisher LSD post-hoc tests.

For the animal studies, a one-way ANOVA and Fisher LSD post-hoc tests were used to detect differences in body, tissue and vascular characteristics. Vasomotor responses were evaluated using repeated-measures ANOVAs to detect differences between experimental groups and drug doses or pressure changes. All values are presented as means ± SE. A value of P ≤ 0.05 was considered statistically significant.

## Additional Information

**How to cite this article**: Delp, M. D. *et al*. Apollo Lunar Astronauts Show Higher Cardiovascular Disease Mortality: Possible Deep Space Radiation Effects on the Vascular Endothelium. *Sci. Rep.* **6**, 29901; doi: 10.1038/srep29901 (2016).

## Supplementary Material

Supplementary Information

## Figures and Tables

**Table 1:** Astronaut characteristics.

AstronautGroups	Selection Year	Age at Selection(years)	Orbital Missions Flown	Time in Space (days)	Age at Death(years)
Non-Flight	1970 ± 1.9 (median 1966)	34.2 ± 1.0	–	–	53.3 ± 3.0
All Flight	1976 ± 2.0 (median 1978)	33.5 ± 0.5	1.9 ± 0.2 (range 1–5)	15.6 ± 1.7 (range 0.2–49.2)	57.5 ± 2.2
Low Earth Orbit	1978 ± 2.1* (median 1978)	33.5 ± 0.6	2.0 ± 0.2 (range 1–5)	15.6 ± 1.8 (range 0.2–49.2)	56.0 ± 2.5
Apollo Lunar	1964 ± 1.0^**†**^(median 1966)	33.4 ± 0.7	1.7 ± 0.4 (range 1–4)	15.2 ± 5.7 (range 6.0–49.2)	65.2 ± 4.0

Values are mean ± SE. *Significantly different from non-flight group, P ≤ 0.05; ^**†**^significantly different from the low Earth orbit group, P ≤ 0.05.

**Table 2:** Proportional mortality rates (%) due to cardiovascular disease, cancer, accidents and all other causes.

	Cardiovascular Disease	Cancer	Accident	Other
Reference Groups				
US Population Ages 55–64, (*n* = 338, 127)	27%	34%	5%	35%
Non-Flight Astronauts, (*n* = 35)	9%*	29%	53%*	9%*
Astronaut Groups				
All Flight Astronauts, (*n* = 42)	17%	31%	43%*	10%*
Low Earth Orbit Astronauts, (*n* = 35)	11%*	31%	49%*	9%*
Apollo Lunar Astronauts, (*n* = 7)	43%†‡	29%	14%^	14%

Values are mean ± SE. *n* = the number of individual deaths per group. *Significantly different from the US population age 55–64 group, P ≤ 0.05; ^**†**^significantly different from non-flight astronaut group, P ≤ 0.05; ^significantly different from the non-flight astronaut group, P ≤ 0.1; ^**‡**^significantly different from the low Earth orbit astronaut group, P ≤ 0.1.

**Table 3:** Tissue and vessel characteristics from control (Con), hindlimb unloaded (HU), total body irradiated (TBI), and TBI plus HU (TBI+HU) mice.

	Con	HU	TBI	TBI+HU
Tissue characteristics:				
Body mass (BM), g	31.0 ± 0.6	31.0 ± 1.0	28.8 ± 0.6	29.7 ± 0.7
Gastrocnemius mass, mg	206.7 ± 4.1	200.1 ± 3.4	200.5 ± 3.6	198.0 ± 5.1
Soleus mass, mg	15.4 ± 0.5	15.8 ± 0.6	14.7 ± 0.6	15.1 ± 0.4
Vessel characteristics:				
Spontaneous tone, %	27 ± 2	25 ± 2	29 ± 2	31 ± 1
Maximal diameter, μm	173 ± 7	171 ± 6	174 ± 5	180 ± 5
Media wall thickness, μm	15 ± 1	15 ± 1	15 ± 1	16 ± 1

Values are mean ± SE. There were no significant differences among groups, P > 0.05.
