# Supplementary material for: Apollo Lunar Astronauts Show Higher Cardiovascular Disease Mortality: Possible Deep Space Radiation Effects on the Vascular Endothelium
Source: Sci Rep. 2016 Jul 28;6:29901. doi: 10.1038/srep29901 (PMC4964660; doi:10.1038/srep29901)

Apollo Lunar Astronauts Show Higher Cardiovascular Disease Mortality: Possible Deep Space Radiation Effects on the Vascular Endothelium

[§]Michael D. Delp, Ph.D.[1], Jacqueline M. Charvat, Ph.D.[2], Charles L. Limoli, Ph.D.[3], Ruth K. Globus, Ph.D.[4] and Payal Ghosh, Ph.D.[1]

[1]Department of Nutrition, Food and Exercise Sciences, Florida State University, Tallahassee, FL 32306,

[2]Wyle Science, Technology and Engineering Group, Johnson Space Center, Houston TX 77058,

[3]Department of Radiation Oncology, University of California Irvine, Irvine, CA 92697, and

[4]Space Biosciences Division, NASA Ames Research Center, Moffett Field, CA 94035

[§]Correspondence: Michael D. Delp, Ph.D.

mdelp@fsu.edu

Supplementary Figure Legends

Figure 1. Effects of HU, TBI, and TBI+HU on vasoconstrictor responses to A) KCl and B) phenylephrine in gastrocnemius muscle feed arteries. Values are mean ± SE. *n* = the number of animals studied. Vasoconstrictor responses are not different among groups.

Figure 2. Effects of HU, TBI, and TBI+HU on A) active myogenic vasoconstrictor responses and B) passive pressure-diameter responses of gastrocnemius muscle feed arteries. Values are mean ± SE. *n* = the number of animals studied. Responses are not different among groups.

Figure 3. Effects of HU, TBI and TBI+HU on A) endothelial nitric oxide synthase (eNOS), B) superoxide dismutase-1 (SOD-1), and C) NADPH oxidase-2 (NOX-2) protein levels in gastrocnemius muscle feed arteries. Values are mean ± SE.

Figure 4. Effects of HU, TBI and TBI+HU on A) endothelial nitric oxide synthase (eNOS), B) superoxide dismutase-1 (SOD-1), and C) NADPH oxidase-2 (NOX-2) protein levels in coronary arteries. Values are mean ± SE.

Supplemental Figure 1

A.

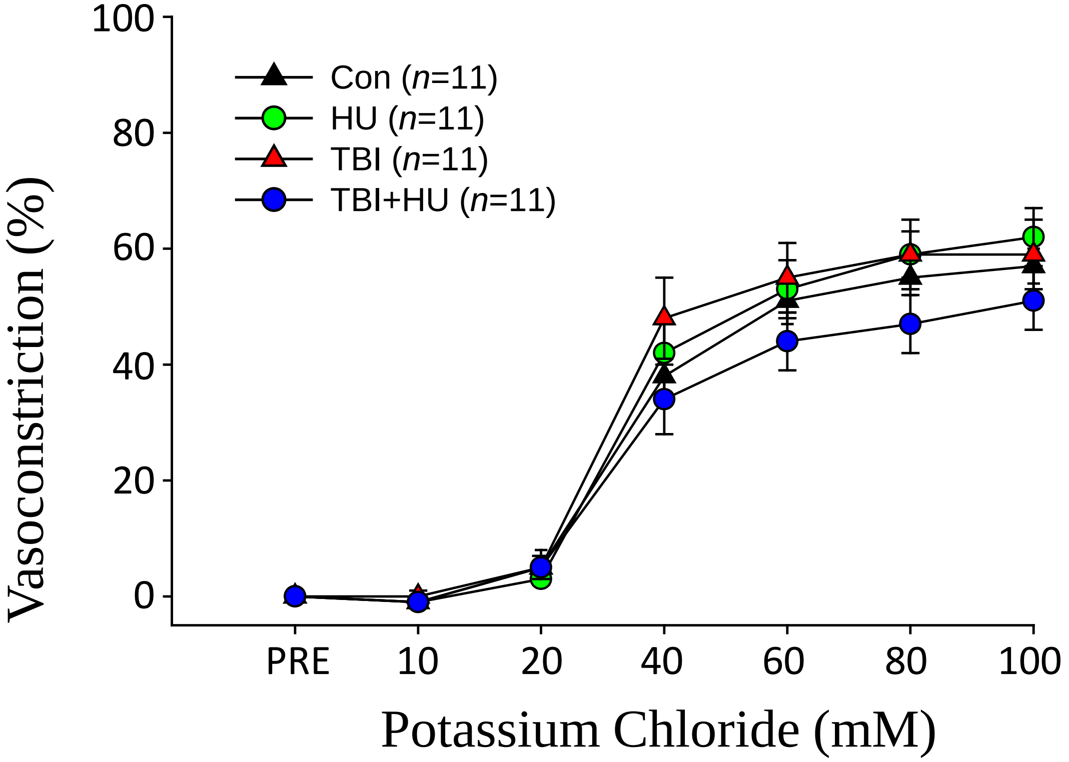

B.

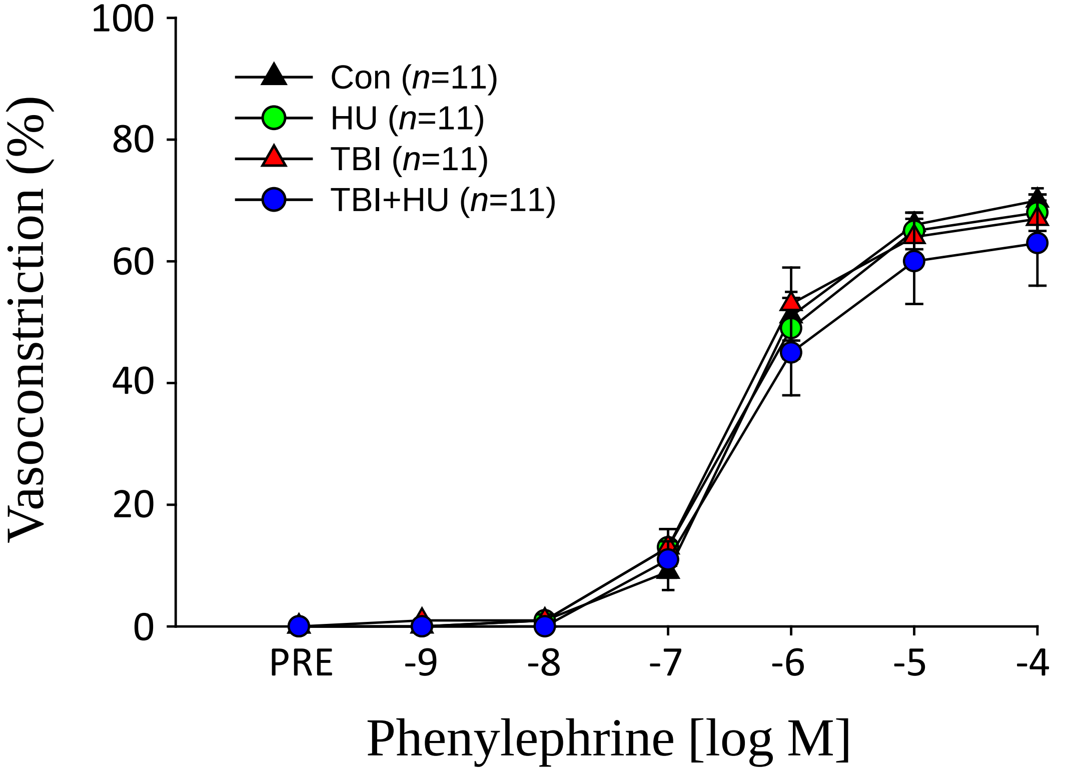

Supplemental Figure 2

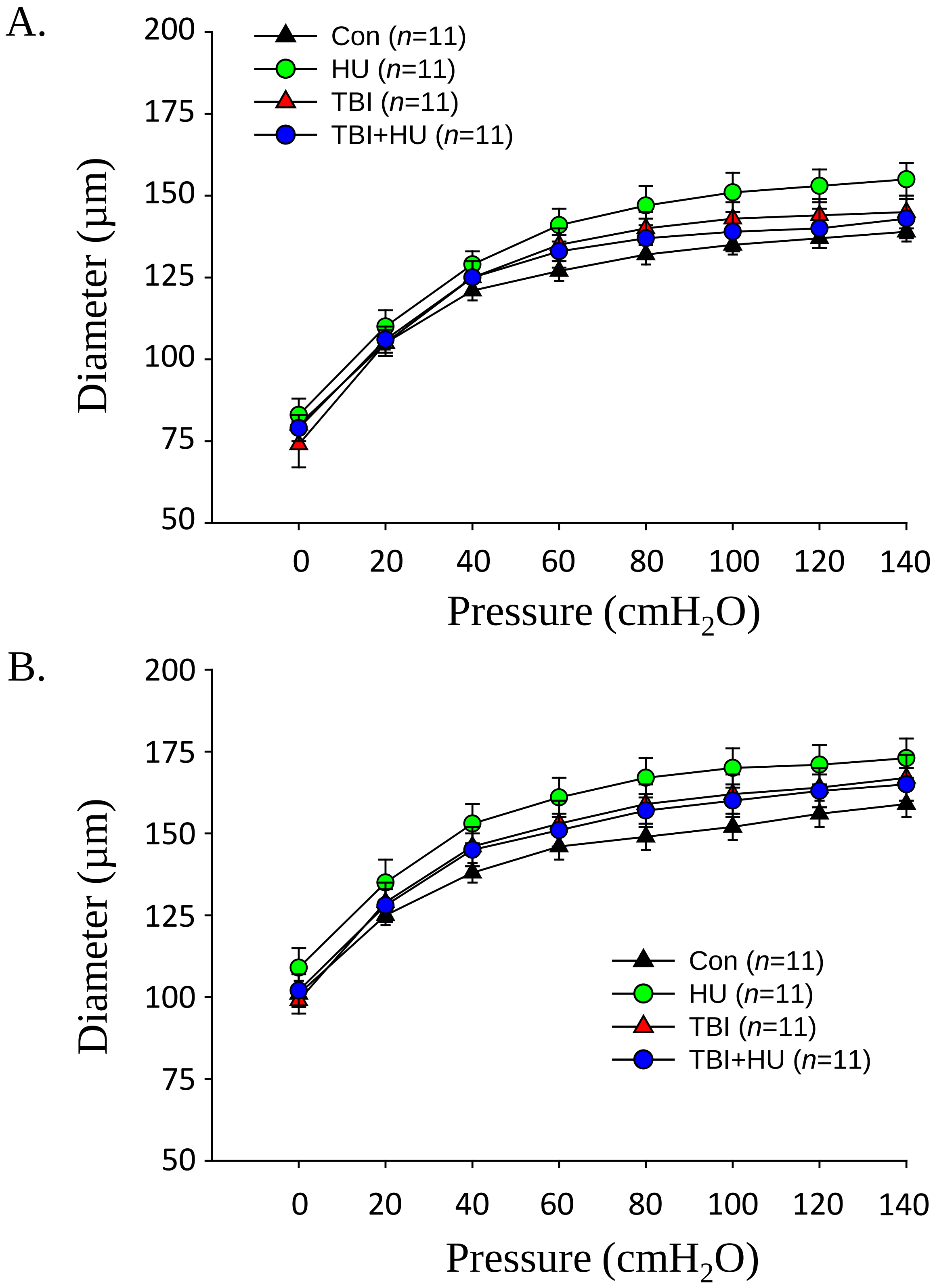

Supplemental Figure 3

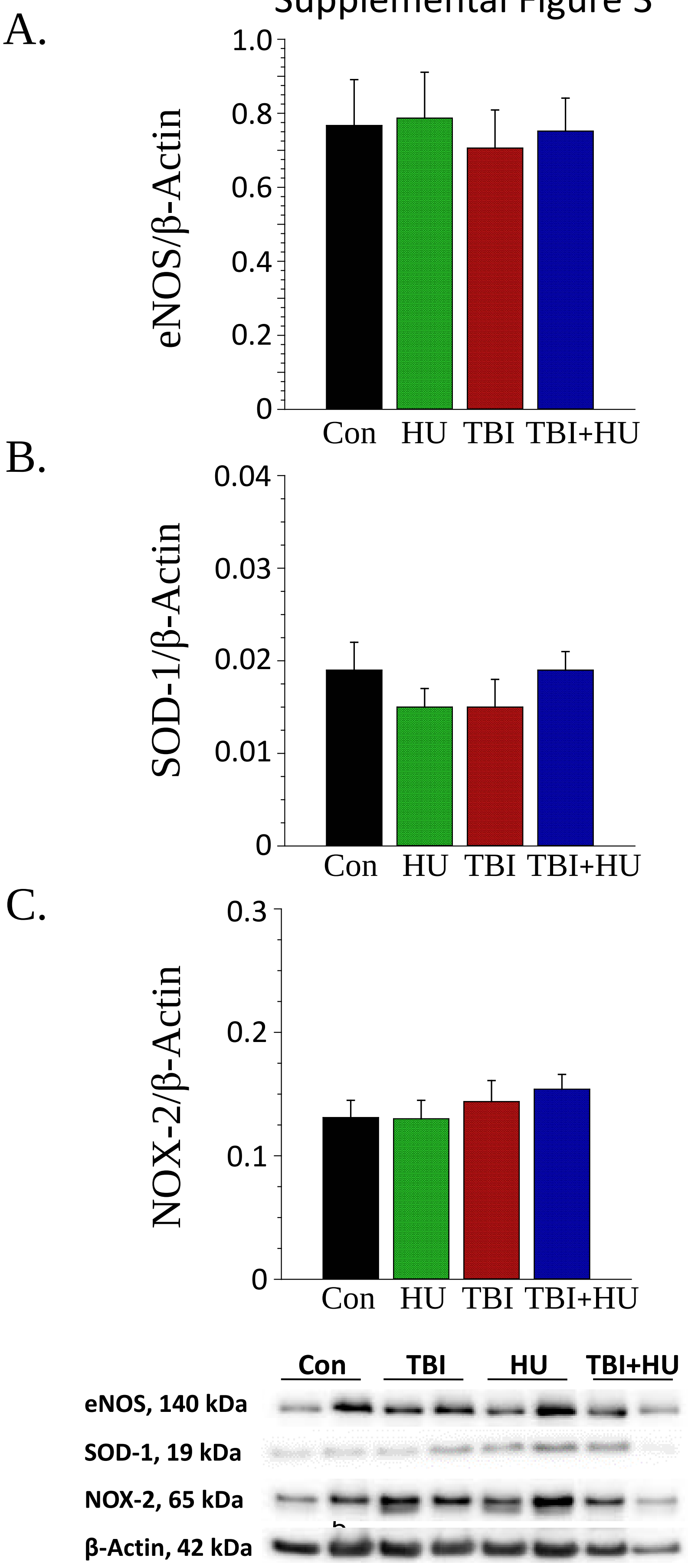

Supplemental Figure 4

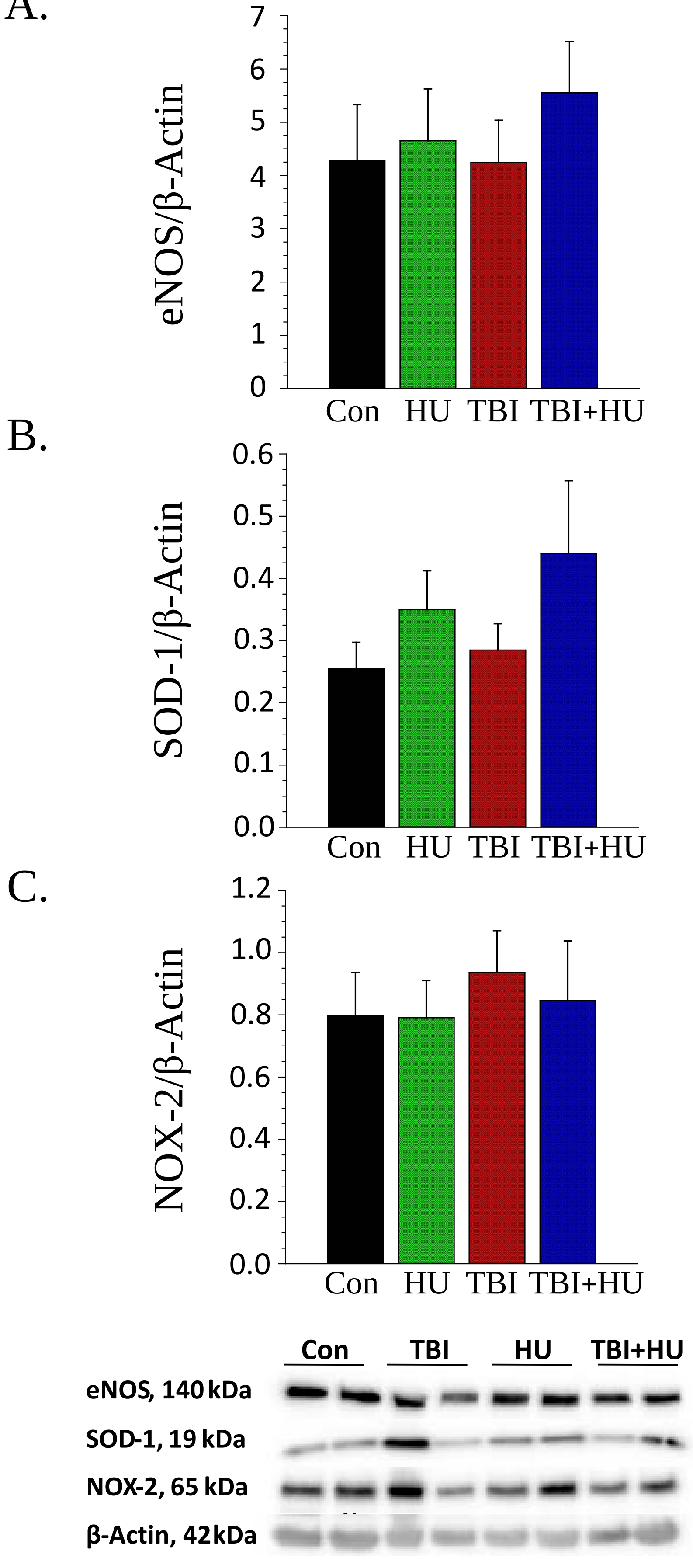

Supplement: Supplementary Information [file srep29901-s1.pdf]
